# Genome-Wide Association Study of Piglet Uniformity and Farrowing Interval

**DOI:** 10.3389/fgene.2017.00194

**Published:** 2017-11-28

**Authors:** Yuan Wang, Xiangdong Ding, Zhen Tan, Chao Ning, Kai Xing, Ting Yang, Yongjie Pan, Dongxiao Sun, Chuduan Wang

**Affiliations:** ^1^Key Laboratory of Animal Genetics and Breeding of Ministry of Agriculture, National Engineering Laboratory of Animal Breeding, College of Animal Science and Technology, China Agricultural University, Beijing, China; ^2^Beijing Shunxin Agriculture Co., Ltd., Beijing, China

**Keywords:** piglet uniformity, farrowing interval, genome-wide association study, pigs, candidates

## Abstract

Piglet uniformity (PU) and farrowing interval (FI) are important reproductive traits related to production and economic profits in the pig industry. However, the genetic architecture of the longitudinal trends of reproductive traits still remains elusive. Herein, we performed a genome-wide association study (GWAS) to detect potential genetic variation and candidate genes underlying the phenotypic records at different parities for PU and FI in a population of 884 Large White pigs. In total, 12 significant SNPs were detected on SSC1, 3, 4, 9, and 14, which collectively explained 1–1.79% of the phenotypic variance for PU from parity 1 to 4, and 2.58–4.11% for FI at different stages. Of these, seven SNPs were located within 16 QTL regions related to swine reproductive traits. One QTL region was associated with birth body weight (related to PU) and contained the peak SNP MARC0040730, and another was associated with plasma FSH concentration (related to FI) and contained the SNP MARC0031325. Finally, some positional candidate genes for PU and FI were identified because of their roles in prenatal skeletal muscle development, fetal energy substrate, pre-implantation, and the expression of mammary gland epithelium. Identification of novel variants and candidate genes will greatly advance our understanding of the genetic mechanisms of PU and FI, and suggest a specific opportunity for improving marker assisted selection or genomic selection in pigs.

## Introduction

In the pig production industry, reproductive traits such as litter size, litter birth weight, and litter mortality play an important role in the development of production and economic profits. In the past few decades, litter size at birth has been treated as an important criterion to evaluate sow productivity (Southwood and Kennedy, 1991; Blasco et al., 1998). However, piglet survival after birth is greatly negatively affected by increasing litter size. Previous genetic studies have shown that piglet uniformity (PU, the within-litter birth weight variability) is positively correlated with piglet mortality (Milligan et al., 2002; Damgaard et al., 2003), and that low birth weight piglets may experience morbidity and mortality. In addition, the farrowing interval (FI, the number of days between two adjacent litters) in a sow's productive life is also an important index in evaluating reproductive ability (Serenius et al., 2003). FI can be considered a comprehensive trait that contains lactation length, weaning to estrus mating interval, and gestation length. Therefore, more attention was focused on PU and FI (Wolf et al., 2005; Cavalcante Neto et al., 2009) than other traits. Both PU and FI are low-heritability traits, where the estimated heritabilities range from 0.05 to 0.12 (Wittenburg et al., 2008; Cavalcante Neto et al., 2009; Zhang et al., 2016). Although we have already made some progress in traditional genetic improvement of reproductive traits, it is still a challenge to understand the biological mechanisms of complex traits (Andersson et al., 2009), which can be an effective alternative basis for breeding programs.

A total of 16,516 QTL associated with 626 different traits in pigs have been reported in previous studies (http://www.animalgenome.org/cgi-bin/QTLdb/SS/index). Among them, 1,416 QTL are associated with reproductive traits. Fifty QTL for PU have been detected across the majority of chromosomes, but there are still no QTL for FI. Although numerous QTL have been detected in domestic animals (King et al., 2003; Holl et al., 2004), these findings are still insufficient because of the low power of linkage analyses and poor resolution in most QTL (Tabor et al., 2002). In recent years, genome-wide association studies (GWAS) have become a powerful strategy for the detection of variation in different traits based on high throughput SNP platforms. It has been widely used in humans (Hindorff et al., 2009; Lauc et al., 2010; Hoffmann et al., 2017) and domestic animals (Petersen et al., 2013; Kominakis et al., 2017). In pigs, many GWAS have been performed on various economically important traits, including immune traits (Luo et al., 2012; Ponsuksili et al., 2016), meat quality traits (Casiró et al., 2017; Verardo et al., 2017), and structural soundness traits (Fan et al., 2011). However, few GWAS have been conducted to assess the genetic architecture of PU and FI. In addition, it is notable that many complex traits undergo dynamic alterations as animals age (Van de Pol and Verhulst, 2006), so the phenotypes of animals at different stages should be used in GWAS to detect genetic variants and increase the statistical power.

In our study, we performed a GWAS on the PU and FI at 4 different stages from first to fourth parity, based on farrowing records using a PorcineSNP80 BeadChip in a Large White pig population. The purpose of this study was to identify the genomic variants and candidate genes that contribute to the phenotypic variability of PU and FI, and promote the improvement of pig breeding programs.

## Materials and methods

### Animals and phenotypes

A total of 884 Large White pigs from the nucleus pig breeding farm of Beijing Shunxin Agriculture Co., Ltd (http://www.000860.com/sxkg/, Beijing, China) were used in our study. We collected blood samples from the jugular vein using the standard procedure of the breeding program, which was approved by the Animal Welfare Committee of China Agricultural University (GB/T 17236–2008).

All pigs had the same genetic background. Farrowing records were collected from parity 1 to 4 during the years 2010 to 2016. Piglet uniformity was defined as the within-litter birth weight variability, usually represented by the coefficient of variation of birth weights within one litter. Farrowing interval was defined as the number of days between two adjacent litters. The rank-based inverse normal transformation of phenotypic values was performed by the function rntransform in the GenABEL package in R (Aulchenko et al., 2007).

### Genotyping and quality control

Genomic DNA was extracted from 1-mL blood samples using TIANamp Genomic DNA kits (Tiangen Biotech, Beijing, China). The quality and quantity of the DNA samples were measured with a NanoDrop™ 2000 (Thermo Fisher Scientific, Waltham, MA, USA). All DNA samples were eligible for genotyping with a ratio of light absorption (A260/280) between 1.8 and 2.0, a concentration >50 ng/μL, and total volume <50 μL. Genotyping was conducted using the Porcine SNP80 BeadChip (GeneSeek, Lincoln, Nebraska, USA) which contained 68,528 SNPs across 18 autosomes and two sex chromosomes. Quality control of the genotype data was carried out using Plink software (Purcell et al., 2007). DNA samples with genotyping of <90% of the markers were removed. The SNPs with call rates < 90%, minor allele frequencies <0.03, Hardy–Weinberg equilibrium (HWE) *P* < 1 × 10^−6^, and the SNPs with no position information and located on the sex chromosomes were also excluded from the dataset. After quality control, the missing genotypes were imputed using Beagle software (Browning and Browning, 2009) based on the remaining SNP genotypes, SNPs with the highest linkage disequilibrium *r*^2^-value larger than 0.3 were retained for further analysis (Yuan et al., 2015).

### Genome-wide association studies

GWAS were implemented independently for PU and FI. From parity 1 to 4, most individuals had more than 1 farrowing record which could be treated as “longitudinal data.” A single-SNP GWAS was performed in ASReml software (Gilmour et al., 2009), and testing was done using a Wald F statistic. The repeatability model was as follows:

Y=1μ+Xb+fm+Zu+Wpe+e

where **Y** is the vector of phenotype values; μ is the overall mean; **b** is the vector of fixed effects including herd, farrowing season, parity, and number of piglets born alive. The fixed effect of number of piglets born alive was only applied in the analysis of PU, where it was classified into four groups: ≤5, 6–11, 12–13, 14–15, and ≥16 (Quesnel et al., 2008); **m** is the incidence vector of SNP genotype scores with values 0, 1, or 2 corresponding to the three genotypes (11, 12, and 22) of the SNP (where two denotes the allele with a minor frequency); *f* is the regression coefficient of phenotypes on SNP genotypes; **u** is the vector of residual polygenetic effects with $u\sim N\;(0,G\sigma_{a}^{2})$, where **G** is the genomic relationship matrix that was constructed through SNP markers (VanRaden, 2008) and $\sigma_{a}^{2}$ is the polygenetic additive variance, compared with pedigree-based relationships, genetic relationship matrices can better remove population structure and control false positives (Kang et al., 2010); **pe** is the vector of random permanent environmental effects with $pe\sim N\;(0,I\sigma_{pe}^{2})$, where $\sigma_{pe}^{2}$ is the permanent environmental variance; **e** is the vector of residual errors with $e\sim N\;(0,I\sigma_{e}^{2})$, where $\sigma_{e}^{2}$ is the residual errors variance; **X**, **Z**, and **W** are the incidence matrices for **b**, **u**, and **pe**, respectively. For most genetic association studies, the effect of any given locus on the trait is very small (Manolio et al., 2009), so we only need to estimate the variance parameters once for each data set, and then apply them to each marker (Kang et al., 2010). We used ASReml software to estimate $\sigma_{a}^{2}$, $\sigma_{pe}^{2}$, and $\sigma_{e}^{2}$ using average information restricted maximum likelihood.

Since Bonferroni correction is overly conservative and may produce false negative results (Johnson et al., 2010), we used the false discovery rate (FDR) (Benjamini and Hochberg, 1995; Weller et al., 1998) to determine the threshold values. FDR was set as 0.01, and the threshold *P*-value was calculated as follows:

$$\begin{array}{l} P=FDR\times \text{n/m} \end{array}$$

where *n* is the number of *P* < 0.01 in the results, and *m* is the total number of SNPs.

The Manhattan plots and quantile-quantile (QQ) plots were drawn by R packages (http://cran.r-project.org/web/packages/gap/index.html). Genomic inflation factor λ was calculated to judge the extent of false positive signals with the estlambda function in the GenABEL packages in R.

GCTA software (Yang et al., 2011) was used to calculate phenotypic variances contributed by significant SNPs for each parity. The genetic relationship matrix (GRM) was created based on genotyped SNPs on chromosomes, and fixed effects (herd, farrowing season, and number of piglets born alive) were treated as covariates to account for potential population structure. The liner mixed model as follows:

$$Y=X\beta +Z_{1}g_{G1}+Z_{2}g_{G2}+e$$

where **Y** is an *n*× 1 vector of phenotypic values for n individuals, **β** is a vector of fixed effects with its incidence matrix **X**, **g**_**G1**_ is a vector of aggregate effects of the selected significant SNPs with its incidence matrix **Z**_**1**_, and $var(g_{G1})=V_{G1}\sigma_{G1}^{2}$ where **V**_**G1**_ represents the selected SNPs-derived GRM with its additive genetic variance $\sigma_{G1}^{2}$, **g**_**G2**_ is a vector of aggregate effects of the other SNPs except the selected significant SNPs and **Z**_**2**_ is an incidence matrix for **g**_**G2**_, and $\text{var}(g_{G2})=V_{G2}\sigma_{G2}^{2}$ where **V**_**G2**_ represents the other SNPs-derived GRM with its additive genetic variance $\sigma_{G2}^{2}$, **e** is the vector of residual errors with $e\sim N\;(0,I\sigma_{e}^{2})$, where $\sigma_{e}^{2}$ is the residual errors variance and **I** is an identity matrix. In this study, the phenotypic variance explained by selected SNPs is defined as $\sigma_{G1}^{2}/\sigma_{p}^{2}$ where $\sigma_{p}^{2}$ is the phenotypic variance.

### Haplotype block analysis

To further detect candidate regions associated with the two traits, we conducted linkage disequilibrium analysis for the chromosomal regions with multiple significant SNPs using Haploview v4.2 (Barrett et al., 2005). A block was defined using the solid spin algorithm by the criteria of Gabriel et al. (2002).

### Gene search and functional annotation

The functional genes containing or near (within 1 Mb) the identified significant SNPs were selected based on the swine genome assembly 10.2 (https://www.ensembl.org/Sus_scrofa/Info/Index). The annotations of genes were carried out using the NCBI database (https://www.ncbi.nlm.nih.gov/) based on the description of gene function and related literatures, simultaneously, gene ontology (GO) analysis was conducted using the DAVID Bioinformatics Resources (https://david.ncifcrf.gov) for PU and FI, respectively. The Fisher's exact test was used to assess the significance of the enriched terms (Dennis et al., 2003; Rivals et al., 2007), and the enriched GO terms with the *P* < 0.05 were selected to explore the genes involved in biological processes (Wang et al., 2015; Xing et al., 2016).

## Results

### Phenotype and SNP data statistics

Descriptive statistics of phenotypes of PU and FI from parity 1 to 4 are shown in Table 1. Neither trait had approximately normal distributions, so the rntransform function in the GenABEL package was used to convert these phenotypes for further analysis.

**Table 1:** Descriptive statistics of PU and FI in a large white population.

**Traits**	**N**	**Units**	**Mean**	**SD**	**Min**	**Max**
PU	2,882	%	13	0.07	1	40
FI	2,263	day	161.68	33.14	127	347

*N, number of phenotypic records in the association study; Mean arithmetic mean, SD, standard deviation, Max, maximum; Min, minimum*.

After quality control, 880 individuals with a genotyping call rates >90% were retained, and 51,727 SNPs were available for the GWAS. The average distance between the neighboring SNPs on each chromosome was calculated (Table S1). The number of SNPs on each chromosome ranged from 1,424 (SSC18) to 5,041 (SSC1), and the adjacent physical distances between them were 30.9 kb (SSC12) to 62.6 kb (SSC1).

### Genome-wide association study

**Figure 1:**
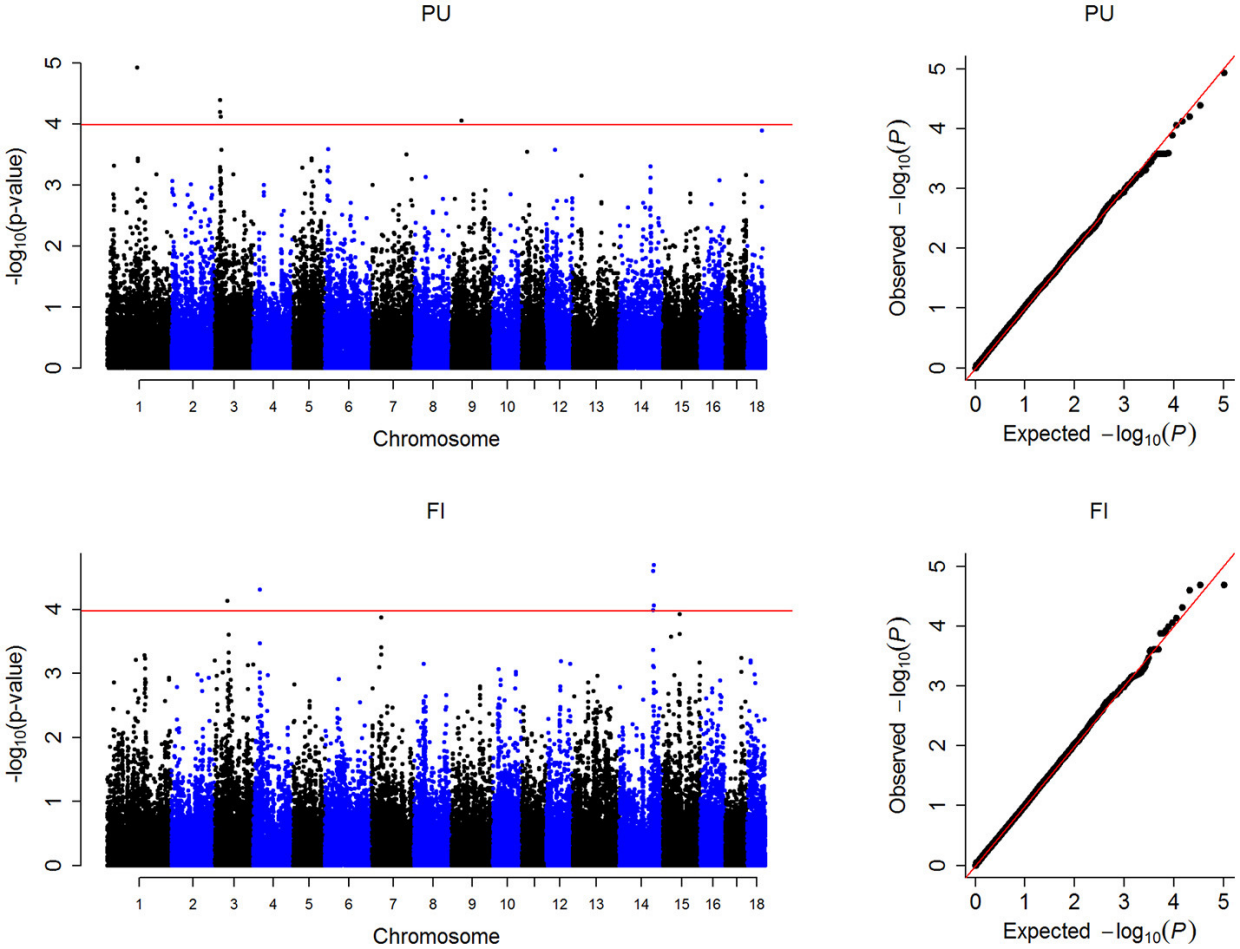
Manhattan plots and Q-Q plots of the observed *P*-values for PU and FI. The Manhattan plots indicate −log_10_ (*P*-values) for genome-wide SNPs (y-axis) plotted against their respective positions on each chromosome (x-axis), the horizontal red lines indicate the thresholds for PU (1.01 × 10^−4^) and FI (1.05 × 10^−4^). The Q-Q plots show the observed −log_10_-transformed *P*-values (y-axis) and the expected −log_10_-transformed *P*-values (x-axis).

In this study, the threshold *P*-values were 1.01 × 10^−4^ and 1.05 × 10^−4^ for PU and FI, respectively. A total of 12 significant SNPs were detected, located on SSC1, 3, 4, 9, and 14 (Figure 1, Table 2). For PU, five significant SNPs were detected, of which three were located on SSC3. For FI, seven significant SNPs were discovered, of which five were located on SSC14. The Manhattan and QQ plots for PU and FI are shown in Figure 1. The genomic inflation factor λ, calculated to judge the extent of false positive signals, was 1.04 for PU and 1.03 for FI.

**Table 2:** All significant SNPs for piglet uniformity and farrowing interval.

**Traits**^**a**^	**SNP**	**Chr**^**b**^	**Position**	***P-*value**	**Nearest/Candidate Gene**^**c**^	**Location(bp)**
PU	MARC0040730	1	108275560	1.2E-05	*LOC100738627*/***SMAD7, LIPG, ACAA2***	Within/**157241, 362855, 594176**
	WU_10.2_3_11397857	3	11397857	6.3E-05	*GTF2IRD1*	23126
	WU_10.2_3_11416624	3	11416624	4.1E-05	*GTF2I*	8020
	ASGA0013487	3	12121767	7.5E-05	*LOC102157744*	Within
	MARC0019308	9	25110615	8.7E-05	*LOC102162346/**UBTFL1***	Within/**495306**
FI	MARC0031325	3	33686233	7.3E-05	*ATF7IP2/**EMP2***	307754/**394825**
	WU_10.2_4_13026957	4	13026957	4.9E-05	*MYC*	243290
	ALGA0081570	14	132795480	2.5E-05	***ADRA2A***	**390234**
	ALGA0081580	14	132843854	1.0E-04	***ADRA2A***	**438608**
	ALGA0081582	14	132860167	8.8E-05	***ADRA2A***	**454921**
	MARC0033692	14	132951697	2.1E-05	***GPAM***	**521618**
	ASGA0066458	14	132972206	2.1E-05	***GPAM***	**501109**

a *PU and FI represent piglet uniformity and farrowing interval*.

b *Pig Chromosome*.

c *The gene name with bold type represents candidate genes with < 1.0 Mb of the SNPs*.

*The bold value means the positions of the candidate genes with bold type*.

### SNP effects

**Figure 2:**
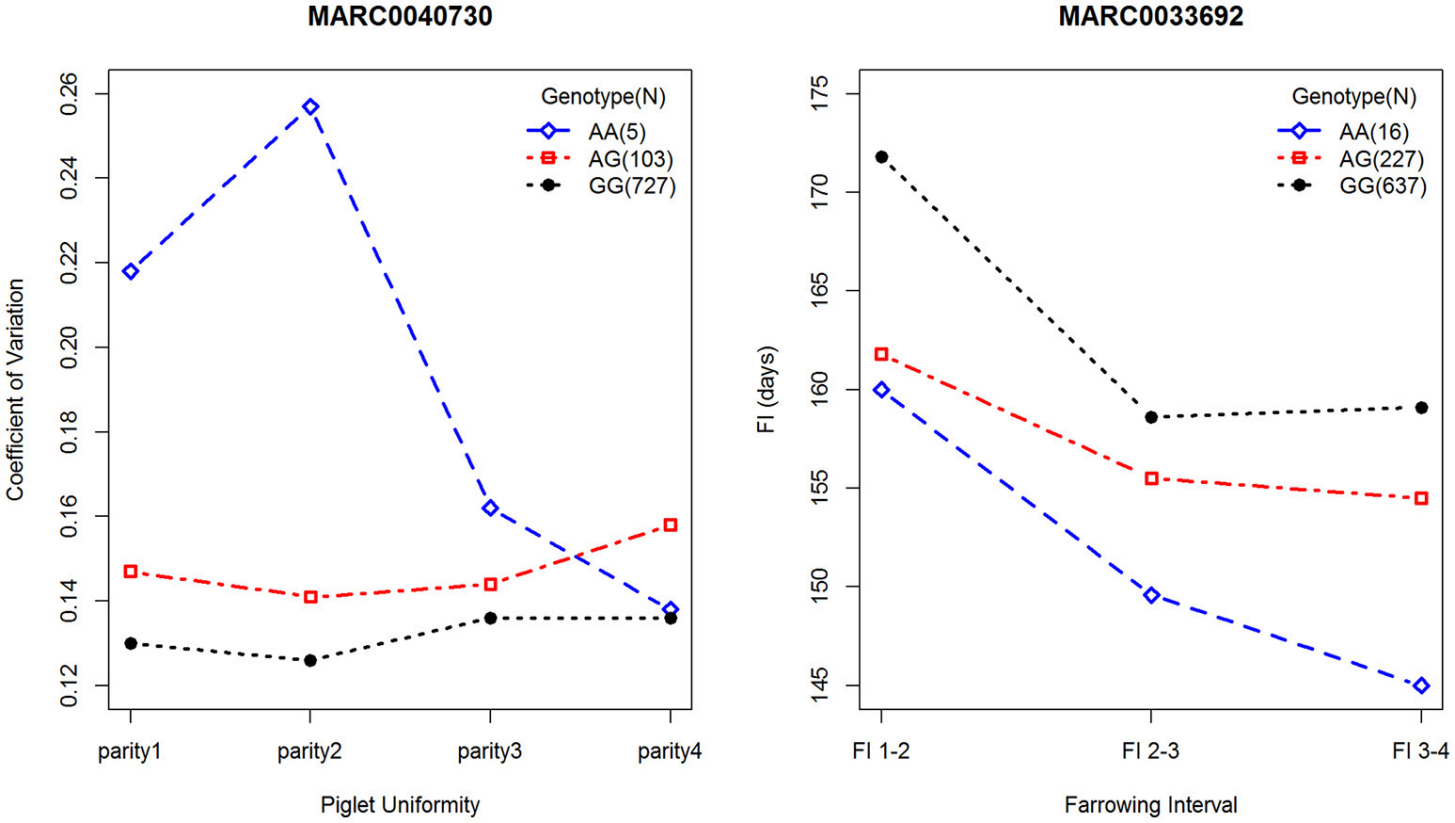
Phenotypic differences contributed by loci of MARC0040730 and MARC0033692. The left plot describes the phenotypes of PU among three genotypes at MARC0040730. The right plot describes the phenotypes of FI among three genotypes at MARC0033692. Blue diamond, red square and black circle represent minor-allele homozygotes, heterozygotes, and major-allele homozygotes, respectively. Number of samples for each genotype is indicated in the top right.

Using GCTA software, the phenotypic variances explained by significant SNPs were estimated for each parity. For PU, five significant SNPs accounted for 1, 1.59, 1.79, and 1.29% of the phenotypic variance from parity 1 to 4, respectively, and the most significant SNP, MARC0040730, located on SSC1, accounted for 0.87–0.95% of the phenotypic variance. Notably, the effect alleles at MARC0040730 were associated with PU at different parities, and the phenotypic differences among the three genotypes at this locus are presented in Figure 2. Along with different parities, each genotype showed different coefficients of variation for birth weights, except for the genotypes AA and GG in the fourth parity. For FI, seven significant SNPs explained 3.17, 4.11, and 2.58% of the phenotypic variance at different parities, respectively. Of note, among them, the peak SNP MARC0033692, located on SSC14, accounted for 1.13–1.18% of the phenotypic variance, and the three genotypes revealed consistent trends for FI at different parities, i.e., the individuals with genotype GG had a longer FI than the other genotypes (Figure 2).

### Haplotype block analysis

**Figure 3:**
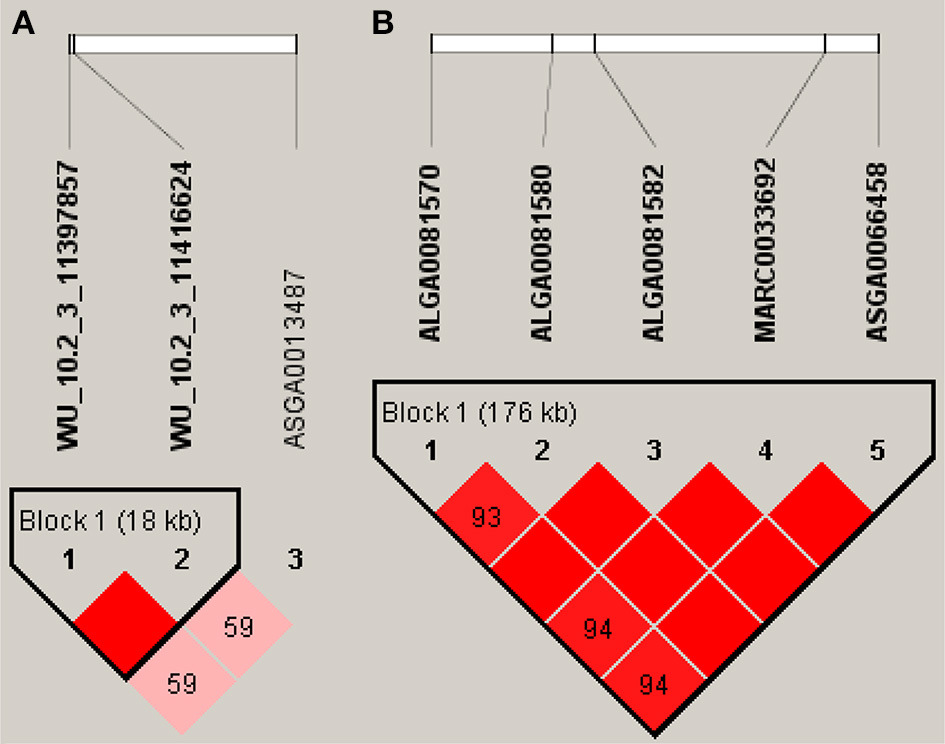
Haplotype blocks for significant SNPs. **(A)** Indicate a haplotype block composed of significant SNPs located on SSC3 for PU. **(B)** Indicate a haplotype block composed of significant SNPs located on SSC14 for FI. The black lines mark the identified blocks.

In our study of FI, we found five significant SNPs located on SSC14, and detected a haplotype block (132.8–132.97 Mb) (Figure 3). The *LOC106506128* gene was located in this region. In particular, the nearest annotated genes of significant SNPs, including the *GPAM* and *ADRA2A* genes, were located downstream (501 Kb) and upstream (390 Kb) of this region, respectively. As for PU, however, relatively few significant SNPs generated haplotype blocks. Two significant SNPs were situated in an 18 Kb block on SSC3 (Figure 3), the *GTF2IRD1* gene located downstream (23.1 Kb) of the region, and *GTF2I* gene located upstream (8 Kb) of the region.

### Candidate genes and function analysis

A total of 61 functional genes that contained or were near (within 1 Mb) the identified significant SNPs were obtained based on the swine genome assembly 10.2 (Table S2). These genes were used to perform GO analysis. Thirteen significant GO terms were identified (Table S3). The most significant GO terms for PU and FI were related to transitions between slow and fast fibers, and cellular iron ion homeostasis, respectively. Considering the genes involved in biological process in DAVID, and functional annotations in the NCBI database and literatures, seven genes (*SMAD7, LIPG, ACAA2, UBTFL1, GPAM, ADAR2A*, and *EMP2*) with biological functions such as prenatal skeletal muscle development, fetal energy substrate, pre-implantation, and the expression of mammary gland epithelium were selected as promising candidates for swine reproductive traits (Table 2). The majority of these were the nearest functional genes to significant SNPs.

## Discussion

Both PU and FI are important indices that can be used to evaluate pig reproductive ability and play crucial roles in production efficiency and economic profits. Thus, it is essential to understand the genetic mechanisms of reproductive performance for future pig breeding programs. GWAS provides an efficient way to detect potential genetic variation and candidate genes in domestic animals (Zhang et al., 2013), especially for some economically valuable traits (Schopen et al., 2011; Sell-Kubiak et al., 2015). In the present study, we conducted a GWAS in a Large White pig population that contained 884 individuals. Our population is larger than that in the PU study of Wang et al. (2016), which consisted of 82 sows. We also made full use of the phenotypic data at four different parities through a repeatability model approach, while some other studies only used the phenotypes of a single parity (Onteru et al., 2012; Schneider et al., 2012). Moreover, this study can detect the genetic variation influencing phenotypic variability over time, and capture parity-independent variation (Smith et al., 2010). Several consistent variants, such as MARC0040730 (located on SSC1) and MARC0033692 (located on SSC14), were found to be associated with PU and FI, respectively, at all parities.

Population stratification is a major factor in false positives in GWAS. Here, through a repeatability model approach, both fixed and random effects were used to adjust the population stratification. In general, a genomic inflation factor λ of <1.05 indicates no population stratification (Price et al., 2010), our values were 1.04 for PU and 1.03 for FI. QQ plots also indicated that we have controlled population stratification, but only a few genomic variants were detected. As we known, quantitative traits are controlled by polygenes (Falconer and Mackay, 1981), and the effect of most given locus on the trait is small. In addition, both PU and FI belong to low-heritability traits, the influence of genetic factor is limited, and the number of samples in our study is still not large enough. Therefore, it's essential to perform studies with a large population and efficient methods for PU and FI in the future.

For PU, considering that the number of piglets born and birth weight were the major influencing factors (Canario et al., 2010), we selected four functional genes near the significant SNPs as important candidates. The *SMAD7* gene, near to the peak SNP MARC0040730, belongs to the SAMD gene family, which has been shown to be a key regulator of transforming growth factor β (TGF-β) (Hayashi et al., 1997). Recent studies have shown that it promotes skeletal muscle cell differentiation (Miyake et al., 2010; Cohen et al., 2015) and follicular development in mice (Gao et al., 2013). Hua et al. (2016) demonstrated that the *SMAD7* gene plays an important role in prenatal skeletal muscle development and promotes weaning weight in pigs. For better fetal growth, the mother must provide sufficient nutrients such as amino acids, glucose, and lipids through the placenta (Brett et al., 2014). Thus, genes associated with energy metabolism may affect fetal growth and piglet birth weight variation. Both *LIPG* and *ACAA2* genes are involved in lipid metabolism, and lipids such as triglycerides and cholesterol play critical roles in fetal growth (Woollett, 2011). The *LIPG* gene encodes a member of the triglyceride lipase family of proteins, which participates in glycerolipid metabolism and transportation of lipids. The *ACAA2* gene encodes the protein that catalyzes the last step in fatty acid β-oxidation (Eaton et al., 1996), and is involved in the fatty acid β-oxidation pathway that can provide fetal energy substrate. Furthermore, fetal lipid deposition is mainly increased in the gestational period (Haggarty, 2002). During the course of reproduction, pre-implantation stages are important for reproductive and stem cell biology (Riaz et al., 2011). The *UBTFL1* gene, also known as *Hmgpi*, is a preimplantation-specific gene and is involved in early development and implantation. Yamada et al. (Yamada et al., 2010) indicated that *Hmgpi* plays a critical role in the earliest stages of mammalian embryonic development.

For FI, which contains lactation length, weaning to estrus mating interval, and gestation length, three functional genes related to the three stages and located near the significant SNPs were selected as potential candidates. The *GPAM* gene encodes a mitochondrial enzyme and catalyzes the initial step in triacylglycerol and phospholipid biosynthesis (Wendel et al., 2009). Knockout studies suggest that the GPAM isoform plays an important role in the development of lactation (Gimeno and Cao, 2008), which is expressed in mammary gland epithelium and is upregulated during lactation. The *ADAR2A* gene encodes a member of the G protein-coupled receptor superfamily, which plays a critical role in the central nervous system. Philipp et al. (2002) showed that α2-adrenoceptors are important regulators of placental structure and function that can help to establish the circulatory system of the placenta between mother and embryo and thus maintain pregnancy. The *EMP2* gene encodes a tetraspan protein that plays an important role in the endometrium and is differentially expressed in the different phases of the estrous cycle (Wadehra et al., 2008). During implantation, *EMP2* is involved in the molecular interactions between the blastocyst-stage embryo and maternal endometrium (Wadehra et al., 2006). Thus, based on functional studies of these genes, we hypothesize that *GPAM, ADAR2A*, and *EMP2* may influence different stages of FI.

Five significant SNPs associated with PU are located on eight QTL regions for reproductive traits, including teat number (Ding et al., 2009), corpus luteum number (Sato et al., 2011), and nonfunctional nipples (Jonas et al., 2008). In particular, the peak SNP MARC0040730, located on a QTL region associated with birth body weight (SSC1, 16.1 to 289.6 Mb) (Guo et al., 2008), and the *SMAD7, LIPG*, and *ACAA2* genes are contained within this region. For FI, we found two significant SNPs, WU_10.2_4_13026957 and MARC0031325, were included within eight reproductive QTLs. One of these QTL regions is related to plasma FSH concentration (SSC3, 21.9 to 38.2 Mb) (Rohrer et al., 2001) and contains both the SNP MARC0031325 and the *EMP2* gene. To summarize, our findings confirmed the importance of these two QTL.

## Conclusion

Our findings provide knowledge on genomic variation and candidate genes that are involved in the genetic mechanisms of PU and FI. The SNPs that are associated with additive genetic variability at different parities can be used as fundamental information in marker assisted selection or genomic selection, which will be helpful in improving pig breeding programs.

## Data accessibility

The genotype and phenotype data of the samples used in the present study are available from the FigShare Repository: https://figshare.com/articles/Genome-wide_Association_Study_of_Piglet_Uniformity_and_Farrowing_Interval/5594230.

## Author contributions

CW and DS designed and supervised the study. YW contributed to genomic DNA extraction and conducted GWAS analysis, XD contributed to population construction and statistical analysis with help from CN, and ZT. KX, ZT, TY, and YP contributed to genomic DNA extraction, phenotypes collection, and samples collection. YW drafted the manuscript, which was critically a remarks by XD, DS, and CW. All authors read and approved the final manuscript.

## Conflict of interest statement

The authors declare that the research was conducted in the absence of any commercial or financial relationships that could be construed as a potential conflict of interest.
