# Supplementary material for: Genome-Wide Association Study of Piglet Uniformity and Farrowing Interval
Source: Front Genet. 2017 Nov 28;8:194. doi: 10.3389/fgene.2017.00194 (PMC5712316; doi:10.3389/fgene.2017.00194)
Supplement: Supplementary file 1 [file Table1.DOCX]

**Table S1. Distributions of SNPs after quality control and the average distance between adjacent SNPs on each chromosome**

| chromosome | SNP number | pysical map(Mb)^a^ | Mb/SNP |
| --- | --- | --- | --- |
| 1 | 5041 | 315.32 | 0.0626 |
| 2 | 3419 | 162.57 | 0.0475 |
| 3 | 3021 | 144.79 | 0.0479 |
| 4 | 3140 | 143.47 | 0.0457 |
| 5 | 2481 | 111.51 | 0.0449 |
| 6 | 3686 | 157.77 | 0.0428 |
| 7 | 3312 | 134.76 | 0.0407 |
| 8 | 2931 | 148.49 | 0.0507 |
| 9 | 3272 | 153.67 | 0.047 |
| 10 | 2280 | 79.1 | 0.0347 |
| 11 | 1923 | 87.69 | 0.0456 |
| 12 | 2059 | 63.59 | 0.0309 |
| 13 | 3684 | 218.64 | 0.0593 |
| 14 | 3412 | 153.85 | 0.0451 |
| 15 | 2951 | 157.68 | 0.0534 |
| 16 | 1944 | 86.9 | 0.0447 |
| 17 | 1747 | 69.7 | 0.0399 |
| 18 | 1424 | 61.22 | 0.043 |

SNP single nucleotide polymorphisms

^a^ The physical size is based on Sus scrofa Build 10.2(http://www.ensembl.org/Sus_scrofa/Info/Index)
